# Serine hydroxymethyltransferase controls blood-meal digestion in the midgut of *Aedes aegypti* mosquitoes

**DOI:** 10.1186/s13071-019-3714-2

**Published:** 2019-09-24

**Authors:** Xuemei Li, Jinyu Yang, Qian Pu, Xinyue Peng, Lili Xu, Shiping Liu

**Affiliations:** 1 State Key Laboratory of Silkworm Genome Biology, Biological Science Research Center, Southwest University, Chongqing, 400715 People’s Republic of China; 2 College of Biotechnology, Southwest University, Chongqing, 400715 People’s Republic of China; 3 College of Life Science, China West Normal University, Nanchong, 637002 People’s Republic of China

**Keywords:** *Aedes aegypti*, Serine hydroxymethyltransferase, Digestive enzyme, Midgut, Blood meal

## Abstract

**Background:**

Female *Aedes aegypti* mosquitoes are vectors of arboviruses that cause diverse diseases of public health significance. Blood protein digestion by midgut proteases provides anautogenous mosquitoes with the nutrients essential for oocyte maturation and egg production. Midgut-specific miR-1174 affects the functions of the midgut through its target gene *serine hydroxymethyltransferase* (*SHMT*). However, less is known about *SHMT*-regulated processes in blood digestion by mosquitoes.

**Methods:**

RNAi of *SHMT* was realized by injection of the double-stranded RNA at 16 h post-eclosion. The expression of *SHMT* at mRNA level and protein level was assayed by real-time PCR and Western blotting, respectively. Statistical analyses were performed with GraphPad7 using Student’s t-test.

**Results:**

Here, we confirmed that digestion of blood was inhibited in *SHMT* RNAi-silenced female *A. aegypti* mosquitoes. Evidence is also presented that all *SHMT*-depleted female mosquitoes lost their flight ability and died within 48 h of a blood meal. Furthermore, most examined digestive enzymes responded differently in their transcriptional expression to RNAi depletion of *SHMT*, with some downregulated, some upregulated and some remaining stable. Phylogenetic analysis showed that transcriptional expression responses to *SHMT* silence were largely unrelated to the sequence similarity between these enzymes.

**Conclusions:**

Overall, this research shows that *SHMT* was expressed at a low level in the midgut of *Aedes aegypti* mosquitoes, but blood-meal digestion was inhibited when *SHMT* was silenced. Transcriptional expressions of different digestive enzymes were affected in response to *SHMT* depletion, suggesting that *SHMT* is required for the blood-meal digestion in the midgut and targeting *SHMT* could provide an effective strategy for vector mosquito population control.

## Background

Female mosquitoes must take a blood meal from vertebrate hosts, including reptiles, birds and mammals, to obtain the proper nutrients for completion of the gonotrophic cycle [1, 2]. The blood-feeding behavior of the mosquito *Aedes aegypti* facilitates the transmission of the most prevalent arboviruses such as the yellow fever virus, dengue virus, chikungunya virus and Zika virus [3–9]. Blood-feeding in mosquitoes induces the production and secretion of many digestive enzymes in the midgut, causing decomposition of blood proteins into peptides and amino acids which serve as essential nutrition sources for vitellogenin biosynthesis and egg development [2, 10–12]. The major classes of digestive enzymes in blood-fed *A. aegypti* female mosquitoes are trypsins, chymotrypsins, aminopeptidases and carboxypeptidases [13, 14]. Over 300 serine protease-like genes were predicted in the *A. aegypti* genome, but only a few serine protease-like trypsins and chymotrypsins have been experimentally confirmed in the midgut [15, 16]. Chymotrypsin is induced by a blood meal in *A. aegypti*, and its protein levels and enzymatic activity remains high during protein digestion [17], but its role during blood-meal digestion remains undetermined. A chymotrypsin-like protease gene, *JHA15*, is transcriptionally activated by juvenile hormone (JH) in the newly emerged female adults but its silencing resulted in no clear phenotype in blood-meal digestion [18]. It is known that the digestion of blood in the midgut of *A. aegypti* mosquitoes can be divided into two phases, early phase one to three hours post-blood-meal (h PBM) and late phase 8–36 h PBM [19]. During the early phase of digestion, the Trypsin 3A1 Precursor AaET (AAEL007818) and female-specific chymotrypsin AaCHYMO (AAEL003060) accumulate [11, 17, 18], but Trypsin-like serine protease AaLT (AAEL013284) and Trypsin 5G1 Precursor Aa5G1 (AAEL013712) begin to be translated by 6–8 h PBM and reach maximal concentrations 24–36 h PBM [20, 21].

Previous work has shown that blood digestion is inhibited when *serine hydroxymethyltransferase* (*SHMT*) is depleted [22]. However, it remains undetermined whether and how *SHMT* regulates the digestive enzymes in the midgut of mosquitoes. As one evolutionarily conserved metabolic enzyme, *SHMT* catalyzes the reversible conversion of L-serine and tetrahydrofolate into glycine and 5,10-methylenetetrahydrofolate [23, 24]. The one-carbon units are raw materials for the synthesis of methylated DNA, and inhibition of *SHMT* activity blocks the biosynthesis of pyrimidine and purine, so SHMT is also an enzyme that links the metabolism of amino acids and nucleotides [25, 26]. SHMT serves as a scaffold protein essential for metabolic complex formation at sites of DNA replication initiation and thus may be even more important determinant of *de novo* thymidylate biosynthesis capacity than its catalytic activity [27]. In humans, SHMT exists in two isozymes in different cellular compartments, commonly termed cytosolic SHMT1 and mitochondrial SHMT2 [28, 29]. SHMT1 preferentially catalyzes serine synthesis, while SHMT2 participates in serine decomposition, thus forming a cyclic one-carbon unit flux between the cytosol and mitochondria [30, 31]. Although *SHMT* has been extensively studied in mammals and some other species, its role in insects is rarely mentioned. Here, our data suggest that *SHMT* controls the blood digestion *via* affecting transcriptional expression levels of diverse digestive enzymes in the midgut of mosquitoes.

## Methods

### Insect breeding and sample collection

The *A. aegypti* (NIH Rockefeller strain) colony was reared at 28 °C and 75% relative humidity under a 12 h light: 12 h dark photoperiod regime. Adult mosquitoes were provided a cotton pad soaked in water (0%) and a pad soaked in 10% sucrose solution before and after a blood meal. The adults were fed rat blood after 3 days of adult eclosion. The blood pellets were removed from the mosquitoes by pinching with forceps under a stereomicroscope before collecting samples. The whole-mount individuals of female *A. aegypti* were collected at 13 time points, namely 6, 12, 24, 36, 48 and 72 h post-eclosion (PE) and 1, 6, 12, 24, 36, 48 and 72 h PBM. Different tissues of adult female mosquitoes were collected before or after a blood meal, including the head (HD), midgut (MG), fat body (FB), ovary (OV) and leftover (LO). All samples were frozen with liquid nitrogen and were separately kept in 300 μl Trizol (for RNA extraction) and stored at – 80 °C.

### Databases and phylogenetic analysis

All sequences of *SHMT* and digestive enzymes were downloaded from VectorBase at https://www.vectorbase.org/. Multi-sequence alignment and phylogenetic analysis were conducted in MEGA X with maximum likelihood method [32].

### RNA extraction, cDNA synthesis and prokaryotic expression

Total RNA was extracted by TRIzol reagent (Invitrogen, Carlsbad, CA, USA) according to the manufacturer’s manual. The first strand of cDNA was synthesized by PrimeScript™ RT reagent Kit with gDNA Eraser (Takara Bio, Shiga, Japan) using 1 μg of total RNA following the manufacturer’s manual. The protein coding region (CDS) of *SHMT* was amplified by PCR using PrimerSTAR Max DNA Polymerase (Takara Bio) with the following PCR program: 98 °C for 5 min; 30 cycles of 98 °C for 10 s, 60 °C for 20 s and 72 °C for 10 s; then 72 °C for 10 min. The primers were designed with Primer Premier 5 and synthesized at Sangon Biotech (Shanghai, China) (Additional file 1: Table S1). The PCR products were purified with an E.Z.N.A.Gel Extraction Kit (Omega Bio-tek, Norcross, GA, USA) following the manufacturer’s manual. After double digestion with restriction endonucleases *Sac*I and *Not*I, the PCR products were cloned into a *pET*-*28a* His-tagged vector. The recombinant plasmids harboring the cloned *SHMT* CDS were then transformed into *Escherichia coli* BL21 (DE3) competent cells (Transgen Biotech, Beijing, China). The transformed recombinant strain *E. coli* BL21 (DE3) was inoculated into LB kanamycin-medium and grown for 2 h at 37 °C in a rotary shaker at 220× *rpm* until OD_600_ was 0.6. Then, prokaryotic expression was induced with 0.1 mmol/l of IPTG at 25 °C for 10 h.

### Purification of recombinant protein and preparation of polyclonal antibody

The host *E. coli* BL21 (DE3) strains above were harvested by centrifugation at 4 °C and 12,000×*g* for 15 min, and were then suspended in binding buffer (20 mM Tris-HCl, 200 mM NaCl, pH 8.0). In order for the cells to rupture sufficiently, the suspended *E. coli* BL21(DE3) strains were repeatedly frozen and thawed in liquid nitrogen 3 times, and were then subjected to intermittent treatment with the ultrasonic cell-break method for 40 min. The recombinant protein SHMT-PA existed in the precipitation, while SHMT-PB was in the supernatant of ultrasound-treated bacterial solution. SHMT-PA precipitation and SHMP-PB were separately collected by centrifugation at 4 °C and 12,000×*g*, and SHMT-PA was then denatured in binding buffer (8 M Urea, 20 mM Tris-HCl, 200 mM NaCl, pH 8.0). Both the SHMT-PB supernatant and denatured SHMT-PA were separately filtered using a 0.45 μm filter membrane. The filtrate was slowly added to Ni ion affinity chromatography column (GE Healthcare, Stockholm, Sweden) with a flow rate of about 4 s/drop. The unbound proteins were rinsed with 40–50 ml of binding buffer (20 mM Tris-HCl, 200 mM NaCl, pH 8.0) with a flow rate of 3–4 s/drop. The His-tag recombinant proteins SHMT-PA and SHMT-PB were then eluted with 100 mM imidazole and 250 mM imidazole, respectively, followed by concentrating through centrifugation at 4 °C and 3,000×*g* in 10 kDa Millipore Amicon Ultra (Merck, Darmstadt, Germany). After purification by Ni-affinity chromatography, SHMT-PB was further purified by AKTA Purifier 100 (GE Healthcare), while SHMT-PA was refolded with gradient PBS buffer (6 M, 4 M, 2 M, 0 M Urea, pH 8.0) at 16 °C. The purified proteins were sent to Wuhan GeneCreate Biological Engineering Co., Ltd (Wuhan, China) to prepare the polyclonal antibodies.

### Double-stranded RNA (dsRNA) synthesis and RNAi knockdown

After analyzing the functional domains of the SHMT with the online software SMART at http://smart.embl-heidelberg.de/, the primers used for *SHMT* RNAi were designed by Primer Premier 5 based on the sequence coding Pfam domain. The protein coding sequences of digestive enzymes were downloaded from VectorBase (https://www.vectorbase.org/organisms/aedes-aegypti) to design the primers for the synthesis of dsRNAs. All primers for RNAi are shown in Additional file 1: Table S1. The dsRNAs for *SHMT* and digestive enzyme genes were synthesized using MEGAscript RNAi Kit (Thermo Fisher Scientific, Waltham, MA, USA) following the manufacturer’s manual. To silence *SHMT* and digestive enzyme genes, mosquitoes at 16 h post-eclosion (PE) were injected with 500 ng of dsRNA of *SHMT* or digestive enzyme genes in 0.5 μl of nuclease-free water. To reveal the dose responses of mosquitoes, we injected different doses of ds*SHMT*, including 50, 100, 200, 400 and 800 ng. Transcript abundance was analyzed by quantitative reverse transcriptase polymerase chain reaction (qRT-PCR), as described below. All dsRNA-injected mosquitoes were maintained on 10% (wt/vol) sucrose solution for 72 h and then a blood meal was given. The ds*EGFP*-injected mosquitoes served as a control. The whole body or tissues of mosquitoes were collected at expected time points for RNA extraction.

### qRT-PCR analysis

The mRNA levels of *SHMT* and digestive enzyme genes were examined by means of qRT-PCR. All primers for qRT-PCR assay were designed with Primer Premier 5 and then synthesized at Sangon Biotech (Shanghai, China) (Additional file 1: Table S1). The cDNA was synthesized with 1μg of total RNA for each sample with Prime Script^™^ RT reagent kit with gDNA Eraser (Takara Bio) according to the manufacturer’s protocol, and was used as a template for qRT-PCR with SYBR^®^ Premix Ex Taq™ II (Takara Bio). The qRT-PCR was performed in a 96-well plate on a real time PCR system, qTOWER2.0 (Analytik Jena, Jena, Germany). The ribosomal protein S7, which is stably expressed in mosquitoes before or after a blood meal [33], served as the baseline control. Unless otherwise stated, all qRT-PCR experiments in this study were performed in triplicate samples with at least three replicates. The relative mRNA expression levels were calculated by using the 2^−ΔΔCT^ method [34].

### Protein extraction and Western blotting analysis

Six female mosquitoes were collected as a whole for the total protein extraction of each sample. The total protein of each sample was prepared in NP-40 Lysis Buffer P0013 (Beyotime, Jiangsu, China). These six mosquitoes were fully ground in 100 μl of lysis buffer and were then incubated in an ice bath for 1 h. After centrifugation at 4 °C, 12,000×*g* for 15 min, the protein in the supernatant was then quantified with a BCA kit P0009 (Beyotime). Next, 50 ng of protein for each sample in 5× SDS loading buffer was denatured at 100 °C in a metal bath for 10 min, and was then loaded onto each gel well, followed by separation using 10% sodium dodecyl sulfate-polyacrylamide gel electrophoresis (SDS-PAGE). After electrophoresis, the proteins on the gel were transferred to the nitrocellulose/PVDF membrane (Roche, Basel, Switzerland) by a semi-dry membrane transfer instrument, Trans-Blot Turbo (Bio-Rad, Hercules, CA, USA). Membranes were blocked with 5% skimmed milk in 20 mM Tris-HCl containing 150 mM NaCl and 0.05% Tween 20 (TBST), then incubated with primary antibodies (1:10,000) against SHMT at room temperature for 2 h. After thoroughly washing with TBST (at least five times), horseradish peroxidase-conjugated secondary antibodies (Beyotime;1:20,000 dilution in TBST) was applied in incubation at room temperature for 1 h. After thoroughly washing with TBST (at least five times), the fluorescence signals were detected using an enhanced chemiluminescence detection kit (Thermo Fisher Scientific), and the photographs were obtained by a Chemiluminescence Imaging System (Clinx Science Instruments, Shanghai, China). The control Anti-TUBULIN (1:20,000) was purchased from Beyotime and the primary rabbit polyclonal anti-SHMT antibody was prepared by Wuhan Gene Create Biological Engineering Co. Ltd. (Wuhan, China).

### Statistical analysis

Statistical analyses were performed using the GraphPad Prism v.7.0 (GraphPad Software, San Diego, CA, USA). The data are presented as means ± SEM of at least three independent samples. Statistical comparisons between groups were performed using one-way ANOVA followed by unpaired Student’s t-test. Differences of *P* < 0.05 were considered statistically significant, and differences of *P* < 0.01 were considered highly statistically significant.

## Results

### Sequence characteristics and expression patterns of *A. aegypti* serine hydroxymethyltransferase

**Fig. 1:**
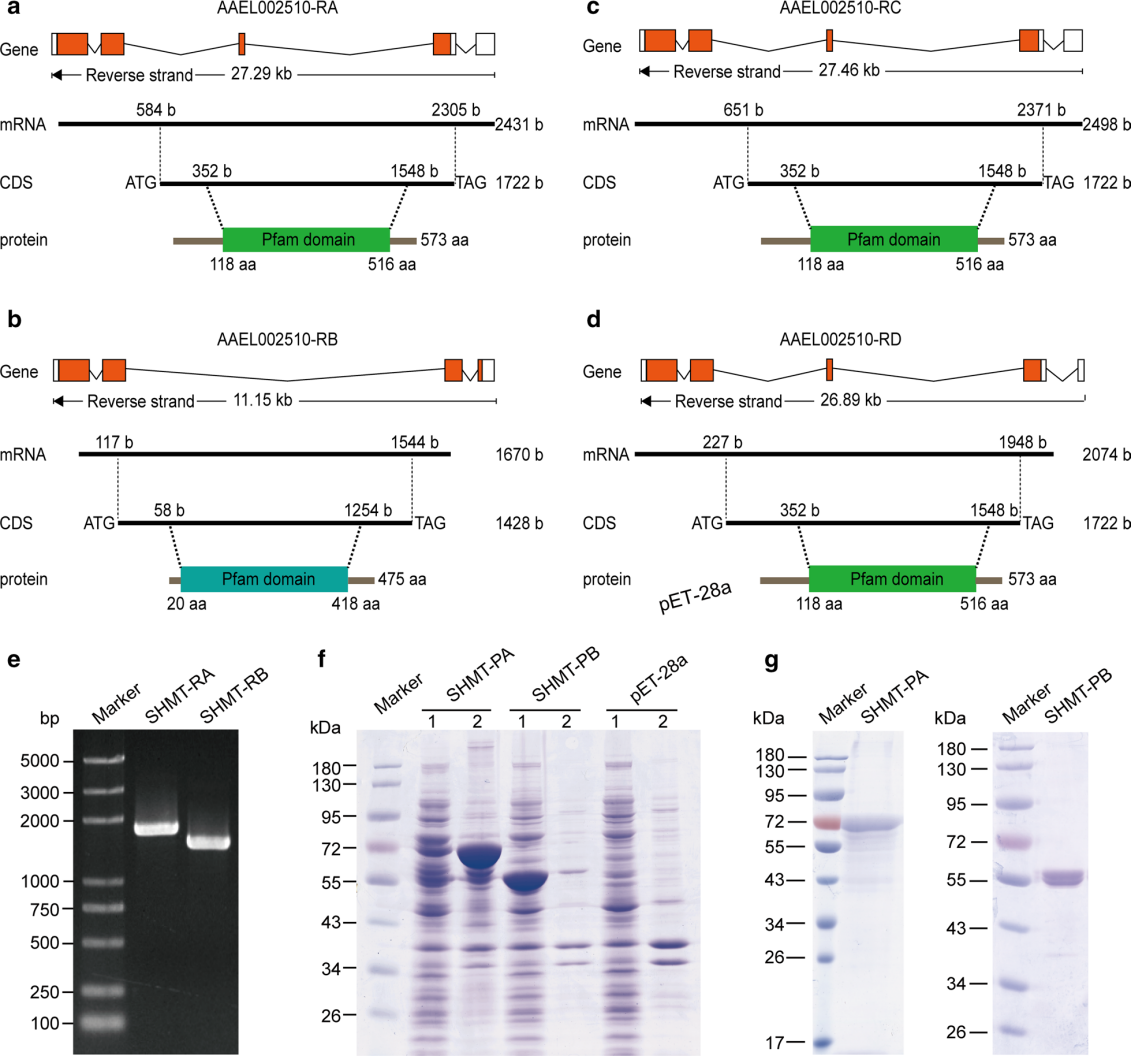
Gene structures, isoforms and prokaryotic expression patterns of *A. aeSHMT*. All sequences were downloaded from VectorBase at https://www.vectorbase.org/organisms/aedes-aegypti. **a**–**d** Schematic diagrams of isoforms A, B, C and D. The square box, black line and red box in the gene structure above represent the exon (mRNA), intron and protein coding region (CDS), respectively. These four *SHMT* isoforms correspond to four different lengths of mRNA and two different lengths of CDS or protein. The Pfam domain was generated by online software Smart at http://smart.embl-heidelberg.de/smart. **e** Cloning of the CDS for SHMT-RA and SHMT-RB. Two different lengths of CDS are shown in the 1% agarose gel as PCR products of SHMT-RA and SHMT-RB. **f** SDS-PAGE showing the SHMT proteins produced by prokaryotic expression. Lanes 1: detection of the supernatant; Lanes 2: detection of the precipitate. **g** SDS-PAGE showing the purified proteins used for the preparation of primary antibody. SHMT-PA was purified only through nickel affinity chromatography, while SHMT-PB was purified through nickel affinity chromatography and by AKTA Purifier 100 (GE Healthcare) (see “Methods”)

**Fig. 2:**
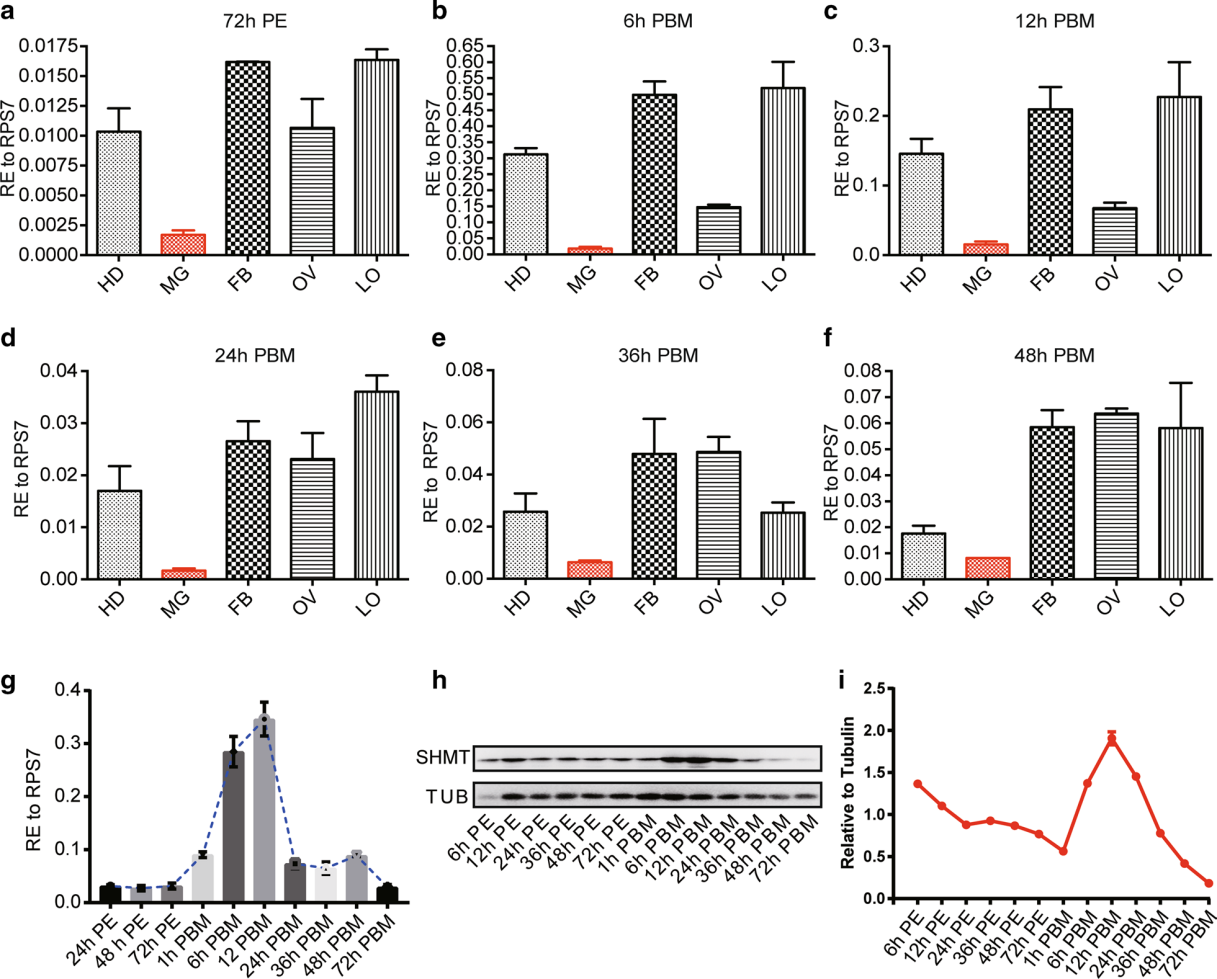
Expression profiles of *SHMT* mRNA and protein in *A. aegypti*. **a**–**f** mRNA levels of *SHMT* in diverse tissues at the following time points: **a** 72 h post-eclosion (PE); **b** 6 h post-blood-meal (PBM); **c** 12 h PBM; **d** 24 h PBM; **e** 36 h PBM; **f** 72 h PBM. **g** mRNA levels of *SHMT* in the whole body of adult females at diverse time points. **h** Western blotting analysis of temporal expression of SHMT in the whole body of adult females. **i** SHMT protein levels in **h** were quantified following normalization to TUBULIN. Nuclear RNA RPS7 was used as a loading control in all qRT-PCR assays (**a**–**g**). The qRT-PCR data are presented as mean ± SEM. TUBLIN was used as the loading control in western blot. *Abbreviations*: RE, relative expression; HD, head; MG, midgut; FB, fat body; OV, ovary; LO, leftover of the whole body

In the latest genome assembly for *A. aegypti* (AaegL5), *SHMT* is located on Chromosome 1:90, 394, 257-90, 421, 721, and has four alternative splicing forms (transcripts), namely AAEL002510-RA, AAEL002510-RB, AAEL002510-RC and AAEL002510-RD, but encodes two isoforms of protein with 573 aa and 475 aa (Fig. 1a–d). The short protein translated from AAEL002510-RB lacks 99 amino acids at the N-end and has seven amino acids different from the longer one encoded by the other three transcripts. The two kinds of protein-coding regions (CDS) were cloned into prokaryotic expression vector and expressed in bacteria (Fig. 1e–g), followed by purifying the proteins to prepare antibodies. We performed qRT-PCR to reveal the transcriptional expression patterns of *SHMT* in diverse tissues of female adults at different time points and found that it was highly expressed in the head, fat body, ovary and leftover tissues but expressed at very low levels in the midgut (Fig. 2a–f); the latter is consistent with our previous evidence [22]. Next, we confirmed that it was expressed at very low levels in the whole body of female mosquitoes before a blood meal (BBM) and quickly increased after a blood meal and even peaked at 12 h post-blood-meal (PBM), but swiftly decreased at 24 h PBM (Fig. 2g). We further examined its temporal expression at the protein level in the whole body (Fig. 2h). Quantified result of the Western blot showed that it was highly expressed at 6 h PE and decreased to basal levels at 1 h PBM, and then quickly increased to the peak level at 12 h PBM (Fig. 2i). Overall, *SHMT* was highly expressed at the early PE phase, then remained at low expression levels throughout the late PE phase but increased rapidly in both transcript and protein levels once the mosquitoes took a blood meal.

### Knockdown of *SHMT* inhibits blood digestion and thus affects ovary development and oviposition

**Fig. 3:**
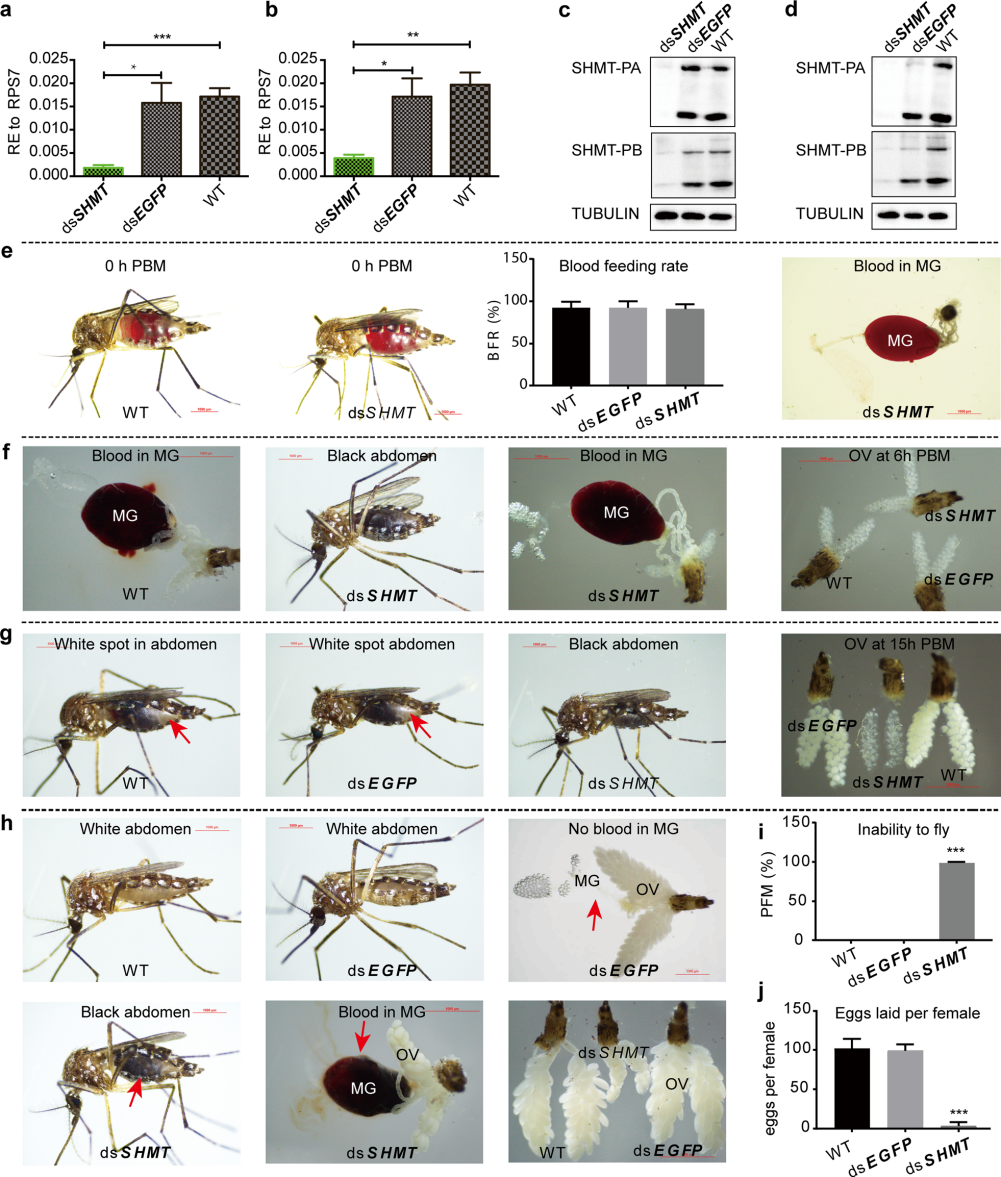
Abnormal phenotypes caused by *SHMT* depletion. **a** qRT-PCR results showing RNAi of *SHMT* at 72 h PE. **b** qRT-PCR results showing RNAi of *SHMT* at 24 h PBM. **c** Western blotting analysis of *SHMT* RNAi at 72 h PE. **d** Western blotting analysis of *SHMT* RNAi at 24 h PBM. **e** RNAi of *SHMT* exerted no effect on blood-feeding. Blood meal was given at 3 d post-injection (PIJ) The wild-type, ds*EGFP*-injected and ds*SHMT*-injected mosquitoes normally sucked the blood which directly entered the midgut. **f** Phenotypes observed at 6 h PBM. The mosquitoes were dissected at 6 h PBM, showing the blood in the midguts of wild-type and ds*SHMT* samples as well as the ovaries of wild-type, ds*EGFP* and ds*SHMT* mosquitoes. **g** Phenotypes observed at 15 h PBM. A white spot appeared at the back of the abdomen for the wild-type and ds*EGFP* samples but the whole abdomen of ds*SHMT* was black. The red arrow shows the white spot in abdomens and growing ovaries. The ovaries are compared between wild-type, ds*EGFP* and ds*SHMT* mosquitoes at 15 h PBM, showing the developmentally delayed eggs in ds*SHMT*. **h** Phenotypes observed at 48 h PBM. The abdomens of wild-type and dsEGFP mosquitoes were all white. The wild-type and ds*EGFP* mosquitoes had no blood in their midguts and the ovaries developed normally. However, the abdomens of *SHMT*-depleted mosquitoes were still black in appearance and had undigested blood in the midgut. Ovaries of WT, ds*EGFP* and ds*SHMT* are compared at 48 h PBM, showing that the ovaries of ds*SHMT* were underdeveloped. **i** The proportion of flightless mosquitoes (PFM). **j** Number of eggs laid per female. The data are shown as the mean ± SEM. *Abbreviations*: dsSHMT, double-stranded RNA of *SHMT*; dsEGFP, double-stranded RNA of EGFP; PBM, post-blood-meal; WT, wild-type; MG, midgut; OV, ovary; BFR, blood-feeding rate; PFM, proportion of flightless mosquitoes. ***P < 0.001

To reveal the function of *SHMT*, we injected 500 ng of ds*SHMT* in 0.5 μl of nuclease-free water into female adult mosquitoes at about 16 h PE. A blood meal was given at 72 h post-injection (h PIJ). The whole-mount individuals and tissues were collected before blood meal about 72 h PE (56 h PIJ) and 24 h PBM for assay by qRT-PCR and Western blot. The results showed that *SHMT* was remarkably decreased at both time points in the mRNA level (Fig. 3a, b) and protein level (Fig. 3c, d). Unlike miR-1174-depleted mosquitoes [22], *SHMT*-silenced mosquitoes displayed no abnormal phenotypes in blood-feeding, and the blood entered the midguts normally (Fig. 3e). At 6 h PBM, the blood in the midguts of *SHMT*-depleted mosquitoes normally turned dark brown as normal, and no obvious abnormal phenotypes could be observed in their ovaries (Fig. 3f). However, abnormal phenotypes were evident at about 15 h PBM (Fig. 3g). For example, the dorsal abdomens of the wild-type and ds*EGFP* controls showed a white spot (red arrow) due to the partial digestion of blood and normal growth of ovaries, whereas the abdomens of the *SHMT*-depleted mosquito were completely black, and the ovaries were about one half the size of the controls. At 48 h PBM, none of the wild-type (WT) or ds*EGFP* controls had blood left in the midguts (red arrow), their abdomens became white in appearance, and their ovaries were growing normally. By contrast, the *SHMT*-depleted mosquitoes still had a majority of undigested blood remaining, and the ovaries were not as developed nor were there as many as in the WT and the ds*EGFP* samples (Fig. 3h). More interestedly, all *SHMT*-depleted mosquitoes were not able to fly within 48 h PBM (Fig. 3i), and almost none of them laid eggs even 4 days after a blood meal (Fig. 3j). The knockdown efficiency of ds*SHMT* was confirmed by qRT-PCR (Fig. 3a, b), showing that 500 ng of ds*SHMT* could significantly silence this gene in the female mosquitoes. Furthermore, different dosages of ds*SHMT* (50, 100, 200, 400 and 800 ng) in 0.5 μl of nuclease-free water each were separately injected into female mosquitoes to compare the flightless time and oviposition (Additional file 2: Figure S1). When each mosquito was injected with 50 ng of ds*SHMT*, all of them could normally fly at 15 h PBM, 10% of them were not able to fly at 24 h PBM and nearly 40% of them were incapable of flying at 40 h PBM; when the injection dosage was increased to 800 ng for each female, about 10% of mosquitoes could not fly within 15 h PBM, and none of them could fly at 40 h PBM (Additional file 2: Figure S1a). The efficiency of different dosages of ds*SHMT* on oviposition was also obvious, i.e. the higher the dose of ds*SHMT* injected, the fewer eggs were laid (Additional file 2: Figure S1b).

### Transcriptional expression of digestive enzymes in the wild-type *A. aegypti* mosquitoes

**Fig. 4:**
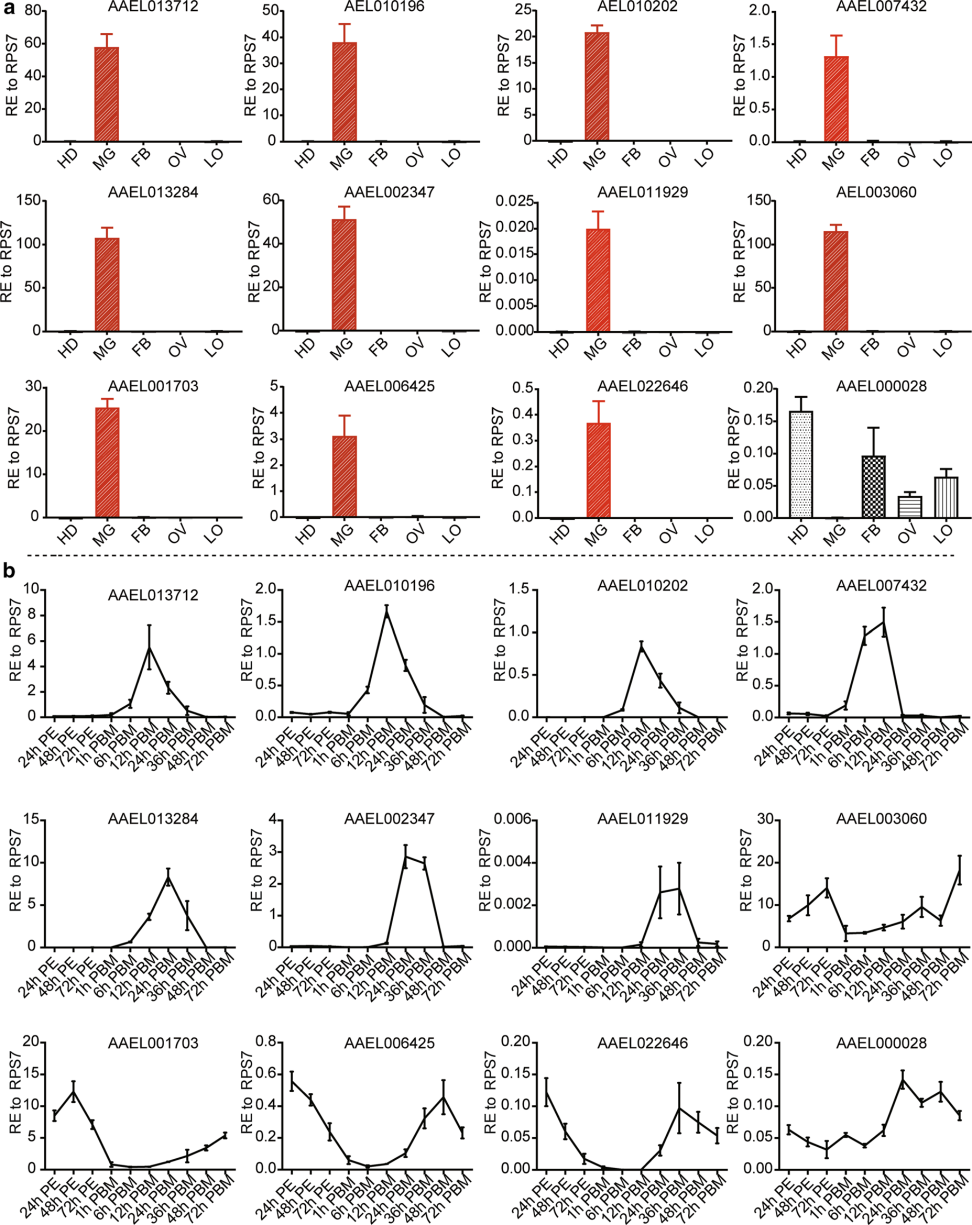
Transcriptional expression profiles of digestive enzymes. Transcriptional expression profiles of 12 known digestive enzyme genes were examined by qRT-PCR (see “Methods”). **a** Spatial expression profiles of digestive enzyme genes at the transcriptional level at 24 h PBM. **b** Temporal expression profiles of digestive enzyme genes at the transcriptional level in adult mosquitoes. AAEL013712, AAEL013284, AAEL006425 are trypsins, AAEL002347, AAEL011929, AAEL003060, AAEL001703, AAEL022646 are chymotrypsins, AAEL010196, AAEL010202, AAEL007432, AAEL000028 are serine proteases. Data are presented as mean ± SEM. *Abbreviations*: HD, head; MG, midgut; FB, fat body; OV, ovary; LO, tissues left over from the whole body

A large family of trypsin-like serine proteases is regarded as the main contributor to the digestion of the blood meal [35, 36]. So far, hundreds of serine protease-like genes have been predicted in the *A. aegypti* genome; however, only a few of them are known to be expressed in the midgut [15, 37–39]. Induced expression of AaET, AaSPVI, AaSPVII and AaLT proteins is not observed until 12 h PBM, with a peak level at 24 h PBM [39]. We speculate that inhibition of blood-meal digestion by *SHMT* RNAi was probably due to abnormal expression of digestive enzymes responding to *SHMT* depletion. To understand the potential effect of *SHMT* RNAi on these enzymes, it is necessary to determine the transcriptional expression patterns of digestive enzymes in wild-type mosquitoes. To this end, we examined the spatial expression of twelve digestive enzymes at 24 h PBM by qRT-PCR (see “Methods”), including three trypsins (AAEL013712, AAEL013284, AAEL006425), five chymotrypsins (AAEL002347, AAEL011929, AAEL003060, AAEL001703, AAEL022646) and four serine proteases (AAEL010196, AAEL010202, AAEL007432, AAEL000028) and found that the expression of eleven enzymes was limited to the midgut, while AAEL000028 was exclusively absent from the midgut (Fig. 4a). AAEL000028 is described as Serine Protease in NCBI (XM_001647815.3), and annotated as Clip-Domain Serine Protease family B in VectorBase. Next, we revealed their temporal expression patterns at a transcriptional level in adults from 24 h PE to 72 h PBM, and found roughly two modes of expression. The first group are those which were weakly expressed or not expressed before a blood meal, but were transcriptionally induced upon blood-feeding to peak levels at 12 or 24 h PBM and then quickly decreased to background levels, including one trypsin (AAEL013712, AAEL013284), three serine proteases (AAEL010196, AAEL010202, AAEL007432) and two chymotrypsins (AAEL002347, AAEL011929). The second group are those which were highly expressed before a blood meal, but dropped to baseline levels promptly after a blood meal and then dramatically returned to high levels at the late PBM phase, including one trypsin (AAEL006425), three chymotrypsins (AAEL003060, AAEL001703, AAEL022646), and one serine protease (AAEL000028) (Fig. 4b). In conclusion, transcriptional expression levels of digestive enzymes in the midguts of *Aedes* mosquitoes responded differently to the blood meal. Admittedly however, our data show mRNA expression profiles for 12 proteases without direct evidence to their involvement in blood-meal digestion. Nevertheless, protease genes for which mRNA expression decreased in response to the blood meal might also be involved in blood-meal digestion because mRNA levels could be high but disappear when translated to protein, especially after a blood meal.

### Transcriptional expression of digestive enzymes responding to depletion of *SHMT*

**Fig. 5:**
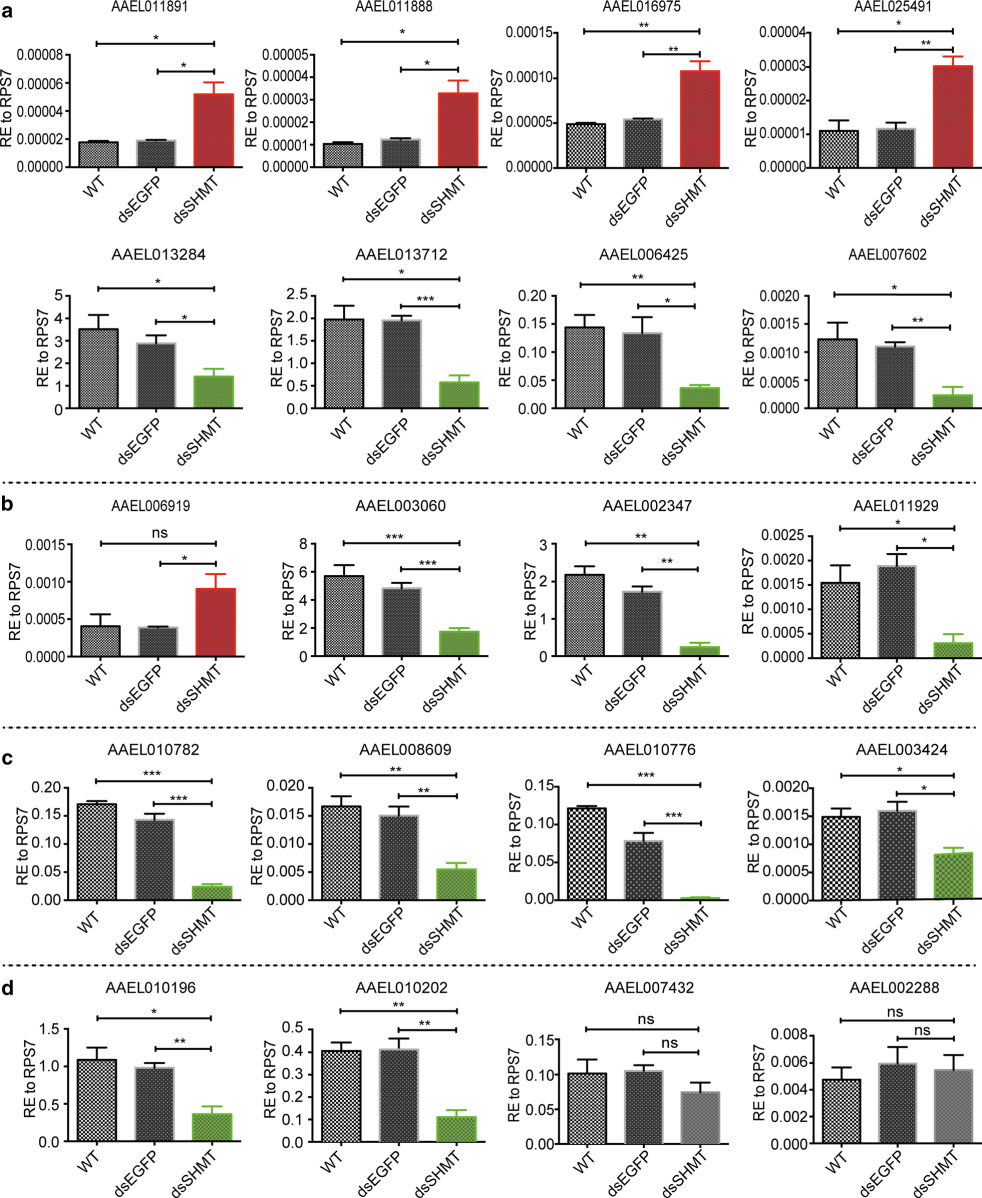
Transcriptional expression responses of digestive enzymes to *SHMT* RNAi. **a** Responses of trypsin to the depletion of *SHMT* by RNAi. **b** Responses of chymotrypsin to the depletion of *SHMT* by RNAi. **c** Responses of carboxyprotease to the depletion of *SHMT* by RNAi. **d** Responses of serine proteases to the depletion of *SHMT* by RNAi. The data are shown as means ± SEM. *P < 0.05; **P < 0.01; ***P < 0.001; ns, not significant

Next, we collected the *SHMT*-depleted whole-mount adult individuals at 24 h PBM and checked the transcriptional expressions of 74 digestive enzymes by qRT-PCR, including 26 trypsins, 26 chymotrypsins, 11 carboxypeptidases and 11 serine proteases (Additional file 1: Table S1 and Additional file 3: Table S2). The results showed that 6 trypsins were significantly increased (AAEL011891, AAEL011888, AAEL016975, AAEL025491, AAEL026329 and AAEL026347), 10 were significantly decreased (AAEL013284, AAEL013712, AAEL006425, AAEL007602, AAEL013703, AAEL007519, AAEL006903, AAEL004543, AAEL011553 and AAEL025114), and 10 remained unchanged (AAEL011889, AAEL006376, AAEL006403, AAEL004885, AAEL007818, AAEL009843, AAEL005611, AAEL024571, AAEL015638 and AAEL000203) (Fig. 5a and Additional file 2: Figure S2).

Among these chymotrypsins, only AAEL006919 was upregulated, 8 were downregulated (AAEL003060, AAEL002347, AAEL011929, AAEL002360, AAEL002177, AAEL011920, AAEL009680 and AAEL024934) and 17 remained unchanged (AAEL011230, AAEL011916, AAEL015294, AAEL017475, AAEL001690, AAEL008784, AAEL006627, AAEL008782, AAEL001703, AAEL011917, AAEL024686, AAEL001690, AAEL004505, AAEL020852, AAEL006383, AAEL007938 and AAEL011922) (Fig. 5b and Additional file 2: Figure S3). Most of the examined carboxypeptidases have been reported to be involved in blood digestion [40]. When the *SHMT* gene was depleted, 6 carboxypeptidases were downregulated (AAEL010782, AAEL010776, AAEL003424, AAEL008609, AAEL001844 and AAEL001855), but 5 showed no significant response (AAEL001840, AAEL008600, AAEL001839, AAEL001863 and AAEL020960) (Fig. 5c and Additional file 2: Figure S4a). Serine proteases, which are characterized by their nucleophilic Ser residue at the active site [41], can be further divided into families and subfamilies, and function as the principle contributors in blood-meal digestion [15]. When *SHMT* was knocked down, the midgut serine proteases AAEL010196 (AaSPVI) and AAEL010202 (AaSPVII) were downregulated, while AAEL007432 (AaSPI), AAEL002288, AAEL000028, AAEL008767, AAEL012558 and AAEL013623 remained unchanged (Fig. 5d and Additional file 2: Figure S4b).

Overall, the digestive enzymes exhibited diverse responses to the depletion of *SHMT* at the transcriptional level. SHMT is known to be involved in the synthesis of dTMP [27, 42], a precursor of dTTP which is essential for nuclear and mitochondrial DNA replication [43]. So, *SHMT* depletion is probably affecting these processes, which in turn is affecting the expression of the proteases in the midgut in an indirect way. Along with regulation of midgut proteases at transcriptional and translational levels when SHMT is knocked down, given its known role in nuclear and mitochondrial DNA replication [27, 44–46], it is likely that DNA replication is also affected.

### Phylogenetic analysis of *A. aegypti* digestive enzymes

**Fig. 6:**
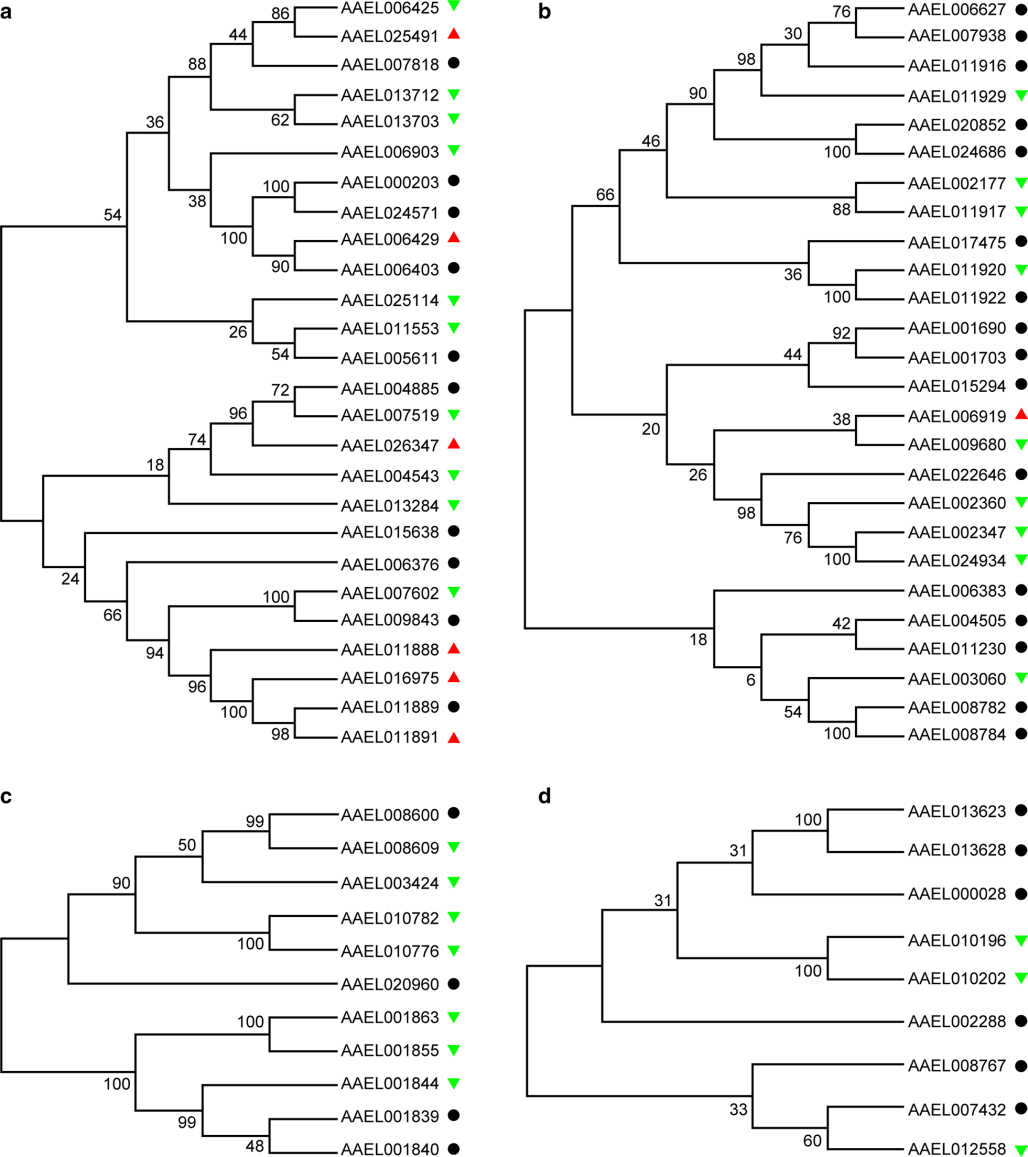
Maximum likelihood phylogenetic trees for digestive enzymes. **a** Trypsins. **b** Chymotrypsins. **c** Carboxyproteases. **d** Serine proteases. The red triangle indicates upregulation by *SHMT* RNAi, the green triangle indicates downregulation, and the black circle indicates unchanged expression

To reveal whether the same expression responses of digestive enzymes are related to their sequence similarity, we downloaded the amino acid sequences of all examined digestive enzymes from VectorBase and performed phylogenetic analysis. For a better comparison, the expression changes are labeled on the phylogenetic tree, with the red triangle meaning upregulated expression, the green triangle representing downregulated expression and the black circle showing unchanged expression. Strikingly, the trypsins were obviously clustered into two large clades, and these three kinds of expression patterns could be observed in each clade (Fig. 6a). The closely clustered trypsins AAEL013712 (Trypsin 5G1 Precursor, Aa5G1) and AAEL013703 (Trypsin) with similar amino acid sequence (identity: 0.3941019) were both downregulated by *SHMT* RNAi. Meanwhile, mRNA expression levels remained unchanged for another sub-clustered AAEL000203 (trypsin-1) and AAEL024571 (trypsin-4), with similar amino acid sequence similarity (identity: 0.4790419) in the *SHMT*-depleted mosquitoes.

By contrast, some members which were clustered in the same sub-clade showed different or even opposite responses. For example, AAEL006429 (trypsin-7) was up-regulated while AAEL006403 (trypsin-7) remained unchanged; these two genes (identity: 0.2893401; Additional file 3: Table S2) are located on different chromosomes. Another pair of closely clustered trypsins AAEL006425 (trypsin-1) and AAEL025491 (trypsin-4-like) (identity: 0.4021448) showed opposite responses to *SHMT* depletion. All of these trypsins are encoded by different loci in the genome and actually have a low degree of homology with each other (Additional file 3: Table S2). Similar to trypsins, the chymotrypsins were organized into two large clades and the members within the same clade did not respond similarly to *SHMT* RNAi (Fig. 6b). A sub-clade member with low sequence similarity (identity: 0.2432432), AAEL006919 (chymotrypsin-1) was upregulated but AAEL009680 (chymotrypsin-2) was downregulated. Although assigned to a sub-clade with high sequence similarity (identity: 0.8248175), AAEL011920 was downregulated but AAEL011922 remained unchanged. However, most members within the same sub-clade with low or high sequence similarity showed exactly the same expression patterns, including AAEL006627 and AAEL007938 (identity: 0.7123746), AAEL020852 and AAEL024686 (identity: 0.9922780), AAEL002177 and AAEL011917 (identity: 0.3891892), AAEL001690 and AAEL001703 (identity: 0.2942708), AEL002347 and AAEL024934 (identity: 0.7894737), AAEL004505 and AAEL011230 (identity: 0.2708861), and AAEL008782 and AAEL008784 (identity: 0.4632768). Therefore, the expression of these chymotrypsins in response to *SHMT* depletion was not directly related to sequence similarity. The similar results could also be observed for carboxypeptidases (Fig. 6c) and serine proteases (Fig. 6d).

## Discussion

Previous studies in other organisms have shown that *SHMT* mainly catalyzes the conversion of serine and tetrahydrofolate to glycine and 5,10-methyl tetrahydrofolate [23, 24]. Increased *SHMT* activity is associated with increased demand for DNA synthesis in rapidly proliferating cells [47]. In recent years, *SHMT* has been shown to be associated with various diseases and has emerged as an important biomarker and drug target [48, 49]. Our previous studies confirmed that *SHMT* is the target gene of mosquito-specific miR-1174. When *SHMT* is downregulated to a certain extent, the abnormal phenotype caused by the depletion of miR-1174 is partially rescued [22], but it remained undetermined whether and how *SHMT* regulates blood digestion, absorption and nutrient signaling in mosquitoes. In this study, we revealed that when the decrease of *SHMT* expression exceeded a certain limit, mosquitoes suffered from blockage of blood digestion, loss of flight and incapability of oviposition. Strikingly, these abnormal phenotypes began to appear in succession about 15 hours after a blood meal. Many factors could cause the failure of blood digestion; a likely possibility is that digestive enzymes are misregulated when *SHMT* is knocked down. At 48 h PBM, the majority of undigested blood remained in the midgut of *SHMT*-depleted mosquitoes, and the egg growth was severely inhibited when compared to that in the WT and the ds*EGFP* samples (Fig. 3h). It seems that blood-meal digestion occurred after ds*SHMT* treatment and enough nutrients could be provided for the formation of ovaries, whereas very few eggs were laid by *SHMT*-depleted mosquitoes (Fig. 3j). *SHMT* RNAi might affect the ovary development and oviposition *via* other processes like DNA replication in the ovary cells since *SHMT* is involved in the *de novo* synthesis of deoxythymidine monophosphate (dTMP) [23, 24], a precursor of deoxythymidine triphosphate (dTTP) [43]. In addition, *SHMT* knockdown recovered the miR-1174 depletion phenotype in mosquitoes [22], so *SHMT* might regulate the egg growth through miRNA regulatory pathway. However, additional evidence will be required for better explanation of the roles of *SHMT* in the ovary development and oviposition.

Previous studies showed that trypsin and chymotrypsin serine proteases produced by midgut epithelial cells might be the main contributors in the blood digestion of some hematophagous dipteran species [35, 36]. Furthermore, dsRNA-mediated knockdown of a single late phase serine protease, including AAEL013284 (AaLT), AAEL010196 (AaSPVI), and AAEL010202 (AaSPVII), significantly retards blood-meal digestion and decreases fecundity, while injection with a mix dsRNA of these protease genes does not show additive or synergistic effect on egg production [39]. All of these digestive enzymes were downregulated in *SHMT*-depleted mosquitoes (Additional file 3: Table S2), logically therefore, their absence might lead to impaired digestion and reproductive disorder. From the expression patterns observed, the digestive enzymes seemed to be negatively or positively regulated by *SHMT* at transcriptional level. We noted the fact that *SHMT* was expressed at low levels in the midgut but highly expressed in other tissues and organs. On the contrary, most digestive enzymes were exclusively upregulated in the midgut. Since the expression of *SHMT* and digestive enzymes is spatially segregated, how does *SHMT* regulate the digestive enzymes in midgut cells? SHMT is known to be involved in the formation of dTTP in DNA synthesis [27, 45, 46], then it seems more likely that transcription of digestive enzyme genes is being affected by SHMT depletion. However, further research is needed to better understand how SMHT RNAi is affecting these digestive enzymes.

The outcome of abnormal phenotype depended on the extent to which *SHMT* is downregulated, but in any case the abnormal phenotype did not appear until 15 hours after a blood meal. Previous studies demonstrated that some early digestive enzymes are quickly synthesized and secreted after a blood meal due to the stimulation of juvenile hormones; these early enzymes preliminarily digest blood and activate the expression of specific transcription factors, enabling some late digestive enzyme genes to be highly expressed 12 hours after a blood meal [50]. After female mosquitoes ingest blood from vertebrate hosts, exopeptidases and endopeptidases are required for digesting blood proteins in the midgut into amino acids, which female mosquitoes use to build yolk proteins. These proteases are not always present in the midgut, and their diverse expression patterns suggest that production of these enzymes is highly regulated in order to meet specific physiological demands at various stages [18]. It is known that the enzyme genes responsible for lipid metabolism in mosquitoes are downregulated at the late juvenile hormone-controlled PE phase and upregulated in the 20-hydroxyecdysone (20E)-mediated PBM phase [51]. *SHMT* might also negatively correlate with the pattern of JH titer while positively correlating with the 20E pulse, and accordingly, when *SHMT* is decreased, the hormone pathway will be blocked, thus resulting in abnormal expressions of various digestive enzymes. However, the evidence for violating these hypotheses remains to be revealed and the regulatory mechanism involved by *SHMT* will not be clearly established until then.

## Conclusions

We examined the transcriptional expression profiles of *SHMT* before and after a blood meal and found that it was highly expressed in the head, fat body, ovary and leftover tissues, was expressed at very low levels in the midgut, and quickly increased in the whole body to the peak level at 12 h PBM. Western blotting analysis showed that the protein levels of *SHMT* also peaked at 12 h PBM in the whole body. Depletion of *SHMT* by RNAi caused severely abnormal phenotypes, including blockage of blood-meal digestion, loss of flight and incapability of oviposition. Strikingly, these abnormal phenotypes began to appear in succession about 15 h after a blood meal. Transcriptional expression of different diverse digestive enzymes was also affected in response to *SHMT* depletion, largely unrelated to the sequence similarity, indicating that *SHMT* might indirectly affect the transcriptional expression of digestive enzymes through its involvement in nuclear and mitochondrial DNA replication [27, 45, 46]. Therefore, we believe we have shown *SHMT* is required for blood-meal digestion in the midgut and targeting *SHMT* could provide an effective strategy for vector mosquito population control.

## Supplementary information

**Additional file 1: Table S1.** All primers used in this study.

**Additional file 2: Figure S1.** Different doses of ds*SHMT* exerted different effect on the flight ability and oviposition of mosquitoes. Different doses of ds*SHMT* (50, 100, 200, 400 and 800 ng) dissolved in 0.5 μl of nuclease-free water were separately injected into mosquitoes at 16 h PE (see “Methods”). The phenotype of mosquitoes was examined at five time points post-blood-meal (PBM), namely 15, 17, 19, 24, 30 and 40 h PBM. **a** Effect on flight ability of mosquitoes. **b** Effect on the oviposition of mosquitoes. **Figure S2.** Transcriptional expression of trypsins responding to *SHMT* RNAi. The data are shown as the mean ± SEM. *P < 0.05; **P < 0.01; ns, not significant. **Figure S3.** Transcriptional expression of chymotrypsins responding to *SHMT* RNAi. All graphs have the same abscissa, i.e. three samples, WT, dsEGFP and dsSHMT. The data are shown as mean ± SEM. *P < 0.05; **P < 0.01; ns, not significant. **Figure S4.** Transcriptional expression of carboxypeptidases and serine proteases responding to *SHMT* RNAi. **a** Transcriptional expression of carboxypeptidases. **b** Transcriptional expression of serine protease. The data are shown as the mean ± SEM. *P < 0.05.

**Additional file 3: Table S2.** Digestion enzymes of *A. aegypti* and their conservative analysis in different species of mosquitoes.

## Data Availability

All data generated or analyzed during this study are included in this published article and its additional files.

## Abbreviations

*SHMT*: serine hydroxymethyltransferase
PE: post-eclosion
BBM: before a blood meal
PBM: post-blood-meal
PIJ: post-injection
HD: head
MG: midgut
FB: fat body
OV: ovary
LO: leftover
CDS: protein coding region
WT: wild-type
qRT-PCR: quantitative reverse transcriptase polymerase chain reaction
20E: 20-hydroxyecdysone
RE: relative expression
dsSHMT: double-stranded RNA of *SHMT*
dsEGFP: double-stranded RNA of EGFP
BFR: blood-feeding rate
PFM: proportion of flightless mosquitoes
SDS-PAGE: sodium dodecyl sulfate-polyacrylamide gel electrophoresis
