# Supplementary material for: Serine hydroxymethyltransferase controls blood-meal digestion in the midgut of Aedes aegypti mosquitoes
Source: Parasit Vectors. 2019 Sep 24;12:460. doi: 10.1186/s13071-019-3714-2 (PMC6757384; doi:10.1186/s13071-019-3714-2)
Supplement: Supplementary file 1 — Additional file 1: Table S1. All primers used in this study. [file 13071_2019_3714_MOESM1_ESM.docx]

**Additional file 1: Table S1.** All primers used in this study

| **Primer** | **sequence** | **use** |
| --- | --- | --- |
| pF-SHMT-RA | 5'CGAGCTC(*Sac*I)-ATGTCGGCCAATATCGTACGTG 3' | CDS |
| pR-SHMT-RA | 5'ATTTGCGGCCGC(*Not*I)-TCTAGAACTCCTCATAGCCTGGCA 3' | CDS |
| pF-SHMT-RB | 5'CGAGCTC (*Sac*I)-ATGCCTCTATGTAATCATAAAAATATGTCC 3' | CDS |
| pR-SHMT-RB | 5'ATTTGCGGCCGC(*Not*I)-TCCTTACAGATCTAAAAACCTCCCAA 3' | CDS |
| pF-SHMT-RA/RB | 5'TAATACGACTCACTATAGGGAGACAGAGATACTACGGCGGCAACGA-3' | RNAi |
| pR-SHMT-RA/RB | 5'AATACGACTCACTATAGGGAGACTGTGTCTGGTAGTCTTTGAACTCTGGC-3’ | RNAi |
| pF-AaLT (AAEL013284) | 5' TAATACGACTCACTATAGGGAGAGTAAACGGACAAACGGCTACCCTC 3' | RNAi |
| pR-AaLT (AAEL013284) | 5' TAATACGACTCACTATAGGGAGATGGCACGGCGATTCCTTGTT 3' | RNAi |
| pF-Aa5G1 (AAEL013712) | 5' TAATACGACTCACTATAGGGAGATGGCTTTGAAGTGCCCGTTG 3' | RNAi |
| pR-Aa5G1 (AAEL013712) | 5' TAATACGACTCACTATAGGGAGAACCACTGACCTCCTTCACCCAAT 3' | RNAi |
| pF-AaSPⅥ (AAEL010196) | 5' TAATACGACTCACTATAGGGAGATATTCTCACAGCAACTTTCTTCGC 3' | RNAi |
| pR-AaSPⅥ (AAEL010196) | 5' TAATACGACTCACTATAGGGAGAAACAATGTCTTCTTACAATCCACTGA 3' | RNAi |
| pF-AaSPⅦ (AAEL010202) | 5' TAATACGACTCACTATAGGGAGACGTATCATCCTTCTGTTAGCGGTA 3' | RNAi |
| pR-AaSPⅦ (AAEL010202) | 5' TAATACGACTCACTATAGGGAGAGCAATGTTCCTAAACTCCACTGAC 3' | RNAi |
| pF-AaCHYMO (AAEL003060) | 5' TAATACGACTCACTATAGGGAGAATCTCGCTGCAGTGGAACTTCAA 3 | RNAi |
| pR-AaCHYMO (AAEL003060) | 5' TAATACGACTCACTATAGGGAGATCACATCCGAGTCCTGTTTCTTTC 3' | RNAi |
| pF-AAEL006425 | 5' TAATACGACTCACTATAGGGAGAAGAGGAAATCGCATTGTGGGTG 3' | RNAi |
| pR-AAEL006425 | 5' TAATACGACTCACTATAGGGAGACCATTGAATAGCACTTGCGTTGAT 3' | RNAi |
| pF-AAEL002347 | 5' TAATACGACTCACTATAGGGAGATTATTTCGTGTGTCCTGATGGCGTC 3' | RNAi |
| pR-AAEL002347 | 5' TAATACGACTCACTATAGGGAGATCGTGATAGTAAGACACTCTGGCGTAA 3' | RNAi |
| pF-EGFP | 5'TAATACGACTCACTATAGGACGTAAACGGCCACAAGTT3' | RNAi |
| pR-EGFP | 5'TAATACGACTCACTATAGGTGCTCAGGTAGTGGTTGTCG3' | RNAi |
|  | F, forward primer; R, reverse primer. Underlined is the promoter of T7. |  |
| **Primers below for *SHMT*** | | |
| q-SHMT-RA/RB_F | 5' GCTCTAAAGGAGGAAGTTCAGGC 3' | qPCR |
| q-SHMT-RA/RB_R | 5' GTATTGGTCATAGCAGCGGACAC 3' | qPCR |
| q-RPS7-F | 5' TCAGTGTACAAGAAGCTGACCGGA 3' | qPCR |
| q-RPS7-R | 5' TTCCGCGCGCGCTCACTTATTAGATT 3' | qPCR |
| **Primers below for 26 Trypsins** | | |
| q-AAEL007818(AaET)-F | 5' ACACCAAGAATGTTTTTGAGATGAA 3' | qPCR |
| q-AAEL007818(AaET)-R | 5' TCCCTGGCAAGAATCCTTACCT 3' | qPCR |
| q-AAEL013284(AaLT)-F | 5' ACTGACCGCAACGGAATACCAC 3' | qPCR |
| q-AAEL013284(AaLT)-R | 5' CCCAGCCACTGACGACTACCTC 3' | qPCR |
| q-AAEL013712(Aa5G1)-F | 5' GATTCAAGGAAGGTGGCAAGGACT 3' | qPCR |
| q-AAEL013712(Aa5G1)-R | 5' AACCACTGACCTCCTTCACCCAA 3' | qPCR |
| q-AAEL004885-F | 5' GCATTGACTTGGGAGCGGAGAT 3' | qPCR |
| q-AAEL004885-R | 5' CGTTGTCTCGGTGACCCAGCTAT 3' | qPCR |
| q-AAEL006376-F | 5' TTTGGACCTGGATTCGGTTTCG 3' | qPCR |
| q-AAEL006376-R | 5' GCTGAATCCGTATCACCGCAAC 3' | qPCR |
| q-AAEL007602-F | 5' GTGGAACCTGGGACCGAACTACTC 3' | qPCR |
| q-AAEL007602-R | 5' ATCCGTTACCGTGGTGTCGTGTT 3' | qPCR |
| q-AAEL013703-F | 5' GAGGATGTTGCGAACGGAAAGC 3' | qPCR |
| q-AAEL013703-R | 5' CTTTTTCTGATTCACCACTGGGACC 3' | qPCR |
| q-AAEL000203-F | 5' ATTCTGACTGCGGCTCATTGTTTAC 3' | qPCR |
| q-AAEL000203-R | 5' AGCCCATAGTCATAATCCTTGCTGTA 3' | qPCR |
| q-AAEL016975-F | 5' ACTGGTGGAATCTCGGCTTGCT 3' | qPCR |
| q-AAEL016975-R | 5' ATCCGCCAATCTGCTGCCTC 3' | qPCR |
| q-AAEL004543-F | 5' CAGGATGGCGACAAATCAGACA 3' | qPCR |
| q-AAEL004543-R | 5' TTCCCGCACATAATTGGGTTTT 3' | qPCR |
| q-AAEL007519-F | 5' GTATGCCCGTGGAAGAAGAAACCTA 3' | qPCR |
| q-AAEL007519-R | 5' TCACTTTGGTTGCGGCACTCAT 3' | qPCR |
| q-AAEL026347-F | 5' TGGCTGGCAAATGTGCTGAAT 3' | qPCR |
| q-AAEL026347-R | 5' GCTACGACACCGCCCACAAT 3' | qPCR |
| q-AAEL011889-F | 5' ACTTCAATGACTTCGTTCAACCTGC 3' | qPCR |
| q-AAEL011889-R | 5' GTTTCACAACCTTCTGTAGGATGGC 3' | qPCR |
| q-AAEL011891-F | 5' TGGCTGTTATGCGTCTGTCAAG 3' | qPCR |
| q-AAEL011891-R | 5' ATGATTGGCTTCACGACCTTTT 3' | qPCR |
| q-AAEL011888-F | 5' TCTCCTTTCGGCTACAACAACTTCA 3' | qPCR |
| q-AAEL011888-R | 5' CATTAGTTGACATTCAGCGTTGGGT 3' | qPCR |
| q-AAEL009843-F | 5' CCTGTTCGGGAGATTCTGGTG 3' | qPCR |
| q-AAEL009843-R | 5' CTTAATCCAATCGGTATAGTAGGAAACA 3' | qPCR |
| q-AAEL006425-F | 5' TCGCTACTGAAGTTGGCTAAAAAAG 3' | qPCR |
| q-AAEL006425-R | 5' TTGTCACGAGATTGGGTTATGCT 3' | qPCR |
| q-AAEL025114-F | 5' CAGCGATTACTATCTGACAAACGACG 3' | qPCR |
| q-AAEL025114-R | 5' CGAGAGCGACGAGTCGTACAGAATC 3' | qPCR |
| q-AAEL015638-F | 5' TGGTGGTGCTGGACAGGAAGGT 3' | qPCR |
| q-AAEL015638-R | 5' GCGGCATTCGTCCAAGTCCA 3' | qPCR |
| q-AAEL011553-F | 5' TTATCTTCGGAGCGGGCATTCA 3' | qPCR |
| q-AAEL011553-R | 5' CAAGACGCTGGGTCCCTGGTAAT 3' | qPCR |
| q-AAEL024571-F | 5' GAGGCGGTGTATTGCTCAGGGT 3' | qPCR |
| q-AAEL024571-R | 5' CCATCCCATCACATCGCATAGG 3' | qPCR |
| q-AAEL025491-F | 5' TTCAAGCCGCTATTGTTCAAGTGTA 3' | qPCR |
| q-AAEL025491-R | 5' GCTCCACGATACAACACCGACAA 3' | qPCR |
| q-AAEL006429-F | 5' GGGCACTGTATCGGAAGGTCATTA 3' | qPCR |
| q-AAEL006429-R | 5' ACTCCAGGTTTGCCTATGGTTGC 3' | qPCR |
| q-AAEL006903-F | 5' GACGGGCAGGTAGACGGGAC 3' | qPCR |
| q-AAEL006903-R | 5' CCTCTGGGAAATACGACATTAGCATA 3' | qPCR |
| q-AAEL006403-F | 5' GACGCAACCAACCCGACCAC 3' | qPCR |
| q-AAEL006403-R | 5' CCACCCGGCTACTTCGCATT 3' | qPCR |
| q-AAEL005611-F | 5' ATTGTCGGAGGTGTAGATGCTGAGA 3' | qPCR |
| q-AAEL005611-R | 5' GCGATTGGCAGAACCAGCAC 3' | qPCR |
| **Primers below for 26 Chymotrypsins** | | |
| q-AAEL003060(AaCHYMO)-F | 5' GCGATGTTACTCTGAGTGGATGG 3' | qPCR |
| q-AAEL003060(AaCHYMO)-R | 5' CGTTGTCCCACAGGGCGTTA 3' | qPCR |
| q-AAEL001703(AaJA15)-F | 5' GTCTGAAGAAGCCTGCGGATAATAAC 3' | qPCR |
| q-AAEL001703(AaJA15)-R | 5' TGCTGTTTCATAGTTTCCTCAATCCA 3' | qPCR |
| q-AAEL008782-F | 5' GCCGAACGGAGATAACCAGACC 3' | qPCR |
| q-AAEL008782-R | 5' CATCGCTAAACTCCAAAGGACGCT 3' | qPCR |
| q-AAEL002177-F | 5' AGATGGGTCGTCACGGCTGGT 3' | qPCR |
| q-AAEL002177-R | 5' TGAATGGCTCCACTACTCGCAGAAC 3' | qPCR |
| q-AAEL006383-F | 5' TTTCGATCTACCCACGAGCATATTG 3' | qPCR |
| q-AAEL006383-R | 5' ATTCGCACCGCATCCAAAGC 3' | qPCR |
| q-AAEL002347-F | 5' TGGCTCTGAAGTTGATATTGGACA 3' | qPCR |
| q-AAEL002347-R | 5' TCGTGATAGTAAGACACTCTGGCGT 3' | qPCR |
| q-AAEL008784-F | 5' TCAGGTGGGCAGAAGCGATG 3' | qPCR |
| q-AAEL008784-R | 5' ATCTTCCAGTTCGGACCACTTGTT 3' | qPCR |
| q-AAEL006919-F | 5' TTGGAGGACAAACGGCTATGAAC 3' | qPCR |
| q-AAEL006919-R | 5' TCGGAAACGATAGACCCACCAC 3' | qPCR |
| q-AAEL004505-F | 5' TTTTCGGATTCGTCAAATGGGAT 3' | qPCR |
| q-AAEL004505-R | 5' GTGATAACCAATGACACTGGAAGGG 3' | qPCR |
| q-AAEL011920-F | 5' GGGTTCTTGGGTGCTGATGGAT 3' | qPCR |
| q-AAEL011920-R | 5' ATGCGAACAAAAACATCTGGGC 3' | qPCR |
| q-AAEL017475-F | 5' CGAAGGTTCACGACAGCAGCAT 3' | qPCR |
| q-AAEL017475-R | 5' ACGATGCGAGGCAACTCTGGT 3' | qPCR |
| q-AAEL011922-F | 5' ATGGAATGCGTGTTGGTGCTT 3' | qPCR |
| q-AAEL011922-R | 5' AATCGCTGAAATACGAACGAAAAC 3' | qPCR |
| q-AAEL011917-F | 5' CGTTATGCGAGTGCGTGTTCC 3' | qPCR |
| q-AAEL011917-R | 5' GGCACTCAGTGTTGGTAATGATGGT 3' | qPCR |
| q-AAEL011916-F | 5' CGTGGCAGGTTTCACTTCGTTC 3' | qPCR |
| q-AAEL011916-R | 5' AACAGATGAGTTCCAACGACGACAC 3' | qPCR |
| q-AAEL011929-F | 5' ATTCAACAATCTGGCTCAAACAA 3' | qPCR |
| q-AAEL011929-R | 5' CAAGGTGATGATGCTGGTGCTC 3' | qPCR |
| q-AAEL020852-F | 5' TGACAATACCATCTGCTCTTCG 3' | qPCR |
| q-AAEL020852-R | 5' GAACACATCTGGGAATCCTTGA 3' | qPCR |
| q-AAEL009680-F | 5' GCGTGGATAGCATCGTGGGAG 3' | qPCR |
| q-AAEL009680-R | 5' GAGCCGACGAGGACCTTGAGAA 3' | qPCR |
| q-AAEL006627-F | 5' GTCGTTGTAGGAACTCATTTGCTG 3' | qPCR |
| q-AAEL006627-R | 5' CAAGAGTGTTGAAGCCGATGAC 3' | qPCR |
| q-AAEL002360-F | 5' TACTGGGAGAAAGAGGTCGGTGCT 3' | qPCR |
| q-AAEL002360-R | 5' TTGCCCGACGATTTCTGCTTG 3' | qPCR |
| q-AAEL024934-F | 5' CAATGGGTTCTAACGGCTGCTC 3' | qPCR |
| q-AAEL024934-R | 5' TTCTTTGCGAGTTTTACCAGGGC 3' | qPCR |
| q-AAEL022646-F | 5' TCGCTACAATCAACTTTTGGACATAAC 3' | qPCR |
| q-AAEL022646-R | 5' TGGTTTATTGTAACGGCTGTGGTGT 3' | qPCR |
| q-AAEL015294-F | 5' ACCAAGTGTCCCTCCAACAAGATG 3' | qPCR |
| q-AAEL015294-R | 5' CACCGTCATTTCGTTGGGTCTTA 3' | qPCR |
| q-AAEL024686-F | 5' GTTCTTCTGTTTGGTTTGGTGGGC 3' | qPCR |
| q-AAEL024686-R | 5' AGCCGAACGCAGCGAGACC 3' | qPCR |
| q-AAEL011230-F | 5' TTTTCTACGAACACGATTTGGCTTTA 3' | qPCR |
| q-AAEL011230-R | 5' TGCGGGCGATTTGCATGTC 3' | qPCR |
| q-AAEL001690-F | 5' AACGAGACGCCGTATGATGAGAA 3' | qPCR |
| q-AAEL001690-R | 5' TCGTAGAACGCCTCACCCAAGT 3' | qPCR |
| q-AAEL007938-F | 5' TGGCTTTGCCCACGCTTCA 3' | qPCR |
| q-AAEL007938-R | 5' CGGCAGCAGTGTTGACGAAGG 3' | qPCR |
| **Primers below for 11 Carboxypeptidases** | | |
| q-AAEL010782-F | 5' GTGGAACTTCTGGAGGGAGGTCAT 3' | qPCR |
| q-AAEL010782-R | 5' TCAGCAATGTTCCGCACTTTAGG 3' | qPCR |
| q-AAEL020960-F | 5' AAGTCACAAAACACTCCTCCATCCTACA 3' | qPCR |
| q-AAEL020960-R | 5' TGAATGCCGCACTCCACGAA 3' | qPCR |
| q-AAEL008600-F | 5' GTGGTCCCTCCTCACAAAGTCG 3' | qPCR |
| q-AAEL008600-R | 5' ACCAGCACGGGCAACACG 3' | qPCR |
| q-AAEL008609-F | 5' CAACGATTGATTGATACGGAACG 3' | qPCR |
| q-AAEL008609-R | 5' CACTGCTCTTACTTCCCTACCTTCAT 3' | qPCR |
| q-AAEL003424-F | 5' GCAAAGTTGAGCGAACGACACG 3' | qPCR |
| q-AAEL003424-R | 5' TCGCAGTTCGTAGCAAAGCACAAT 3' | qPCR |
| q-AAEL010776-F | 5' GGAACACTTACGACGCCATCTAC 3' | qPCR |
| q-AAEL010776-R | 5' TAGAATGAACCCACCCGATGAC 3' | qPCR |
| q-AAEL001863-F | 5' TACGCTGCGGCTGGTGGAA 3' | qPCR |
| q-AAEL001863-R | 5' CATTGCTTTGATGCCCACCCA 3' | qPCR |
| q-AAEL001839-F | 5' TATTGCTATGGGTGAAAAGGCTAAGG 3' | qPCR |
| q-AAEL001839-R | 5' CCTCCGAGTCCCAAAGCGTAAT 3' | qPCR |
| q-AAEL001840-F | 5' GAAGTGAACCGATTGTGGAGGAAG 3' | qPCR |
| q-AAEL001840-R | 5' GCGTCTCCGATTCCGTTGC 3' | qPCR |
| q-AAEL001844-F | 5' GTCGCAACACCAAGCAAGATGATAC 3' | qPCR |
| q-AAEL001844-R | 5' CAACAACTCCCGTGGCACTGAT 3' | qPCR |
| q-AAEL001855-F | 5' AAACAGTCACCATCGGACAAAGT 3' | qPCR |
| q-AAEL001855-R | 5' GTAGCCGTCTGGATTCACAAGC 3' | qPCR |
| **Primers below for 11 Serine proteases** | | |
| q-AAEL007432 (AaSPⅠ)-F | 5' TGAGGTCTACACCAGTGGATACGG 3' | qPCR |
| q-AAEL007432 (AaSPⅠ)-R | 5' ACATCGCTGACACAGTTGGGTTC 3' | qPCR |
| q-AAEL008093 (AaSPⅡ)-F | Same as AAEL013623 and deleted in AaegL5 | qPCR |
| q-AAEL008093 (AaSPⅡ)-R | Same as AAEL013623 and deleted in AaegL5 | qPCR |
| q-AAEL013623 (AaSPⅢ)-F | 5' CGTGGCAGCATCGGTGGAC 3' | qPCR |
| q-AAEL013623 (AaSPⅢ)-R | 5' AGTGGGCAGCCGTCAGAACC 3' | qPCR |
| q-AAEL013628 (AaSPⅣ)-F | Same as AAEL013623 | qPCR |
| q-AAEL013628 (AaSPⅣ)-R | Same as AAEL013623 | qPCR |
| q-AAEL008085 (AaSPⅤ)-F | Same as AAEL013623 and deleted in AaegL5 | qPCR |
| q-AAEL008085 (AaSPⅤ)-R | Same as AAEL013623 and deleted in AaegL5 | qPCR |
| q-AAEL010196 (AaSPⅥ)-F | 5' TAAGTTGCCTCAGAAGGACGCT 3' | qPCR |
| q-AAEL010196 (AaSPⅥ)-R | 5' AAGTAAGCCTTGTGGCATTCCTC 3' | qPCR |
| q-AAEL010202 (AaSPⅦ)-F | 5' ATCCACCATTGACTACGATTTTTGTTTG 3' | qPCR |
| q-AAEL010202 (AaSPⅦ)-R | 5' GTTTTGAGTATATCCCCAGCCCGA 3' | qPCR |
| q-AAEL002288-F | 5' GCCAATGTTCTGGACACGCTTA 3' | qPCR |
| q-AAEL002288-R | 5' CGTAGTGTTCGTGGACAATCATCT 3' | qPCR |
| q-AAEL008767-F | 5' GGAAGTGACGCTGATAGGGTGG 3' | qPCR |
| q-AAEL008767-R | 5' GGAGTCACCATTACATTGCCCTC 3' | qPCR |
| q-AAEL000028-F | 5' GTTCAGTTTTCGTCGTTCATTTCTC 3' | qPCR |
| q-AAEL000028-R | 5' CTCCCGATGGTCTGTATGTGTTG 3' | qPCR |
| q-AAEL012558-F | 5' AATCCGTCAGTCTCGTATCTCCG 3' | qPCR |
| q-AAEL012558-R | 5' ACGAACCGAGCGGAAATCAA 3' | qPCR |
