# Supplementary material for: Serine hydroxymethyltransferase controls blood-meal digestion in the midgut of Aedes aegypti mosquitoes
Source: Parasit Vectors. 2019 Sep 24;12:460. doi: 10.1186/s13071-019-3714-2 (PMC6757384; doi:10.1186/s13071-019-3714-2)
Supplement: Supplementary file 2 — Additional file 2: Figure S1. Different doses of dsSHMT exerted different effect on the flight ability and oviposition of mosquitoes. Different doses of dsSHMT (50, 100, 200, 400 and 800 ng) dissolved in 0.5 μl of nuclease-free water were separately injected into mosquitoes at 16 h PE (see “Methods”). The phenotype of mosquitoes was examined at five time points post-blood-meal (PBM), namely 15, 17, 19, 24, 30 and 40 h PBM. a Effect on flight ability of mosquitoes. b Effect on the oviposition of mosquitoes. Figure S2. Transcriptional expression of trypsins responding to SHMT RNAi. The data are shown as the mean ± SEM. *P < 0.05; **P < 0.01; ns, not significant. Figure S3. Transcriptional expression of chymotrypsins responding to SHMT RNAi. All graphs have the same abscissa, i.e. three samples, WT, dsEGFP and dsSHMT. The data are shown as mean ± SEM. *P < 0.05; **P < 0.01; ns, not significant. Figure S4. Transcriptional expression of carboxypeptidases and serine proteases responding to SHMT RNAi. a Transcriptional expression of carboxypeptidases. b Transcriptional expression of serine protease. The data are shown as the mean ± SEM. *P < 0.05. [file 13071_2019_3714_MOESM2_ESM.docx]

**Additional file 2.**


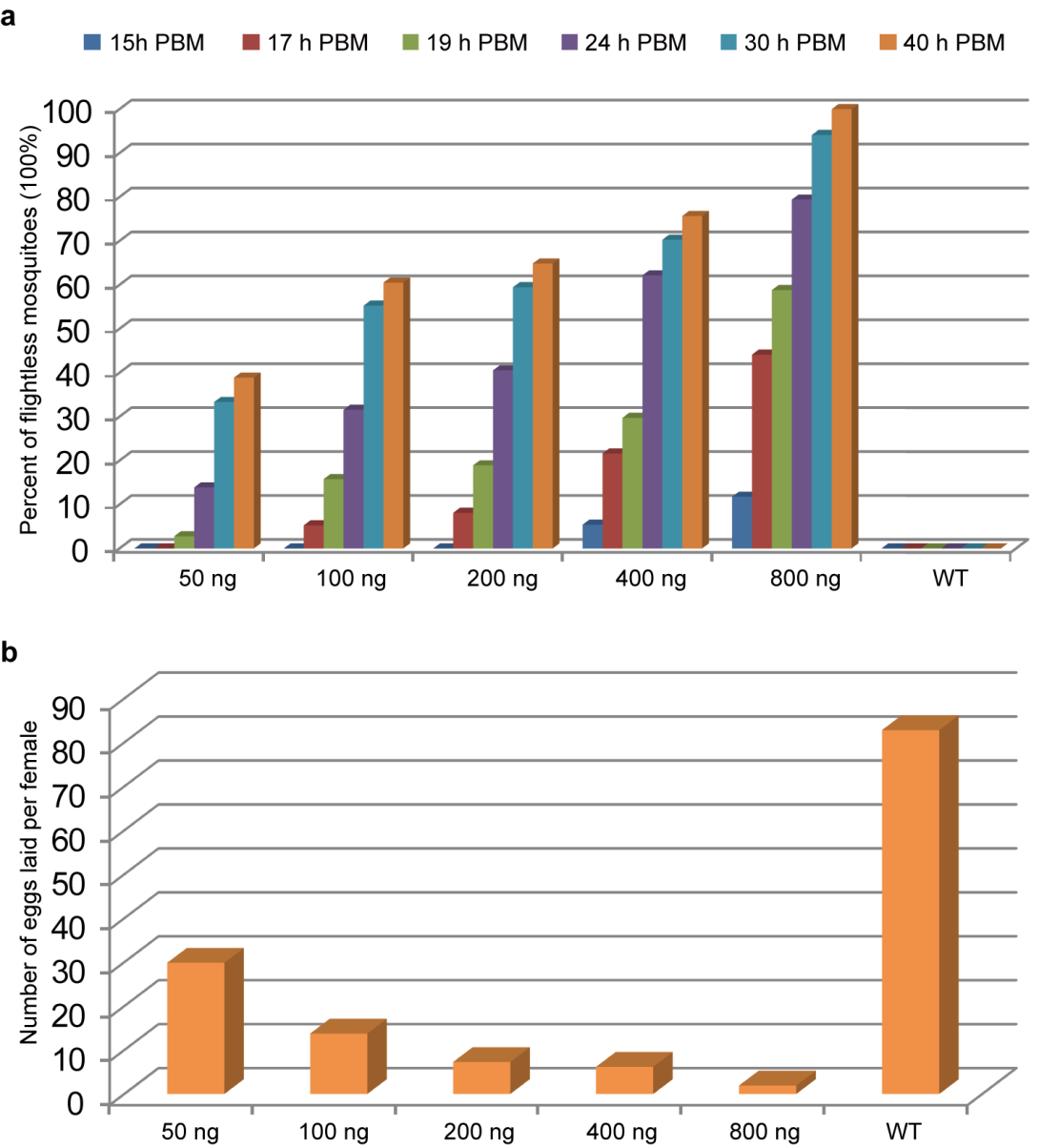


**Figure S1.** Different doses of ds*SHMT* exerted different effect on the flight ability and oviposition of mosquitoes. Different doses of ds*SHMT* (50, 100, 200, 400 and 800 ng) dissolved in 0.5 μl of nuclease-free water were separately injected into mosquitoes at 16 h PE (see Methods). The phenotype of mosquitoes was examined at five time points post-blood-meal (PBM), namely 15, 17, 19, 24, 30 and 40 h PBM. **a** Effect on flight ability of mosquitoes. **b** Effect on the oviposition of mosquitoes.


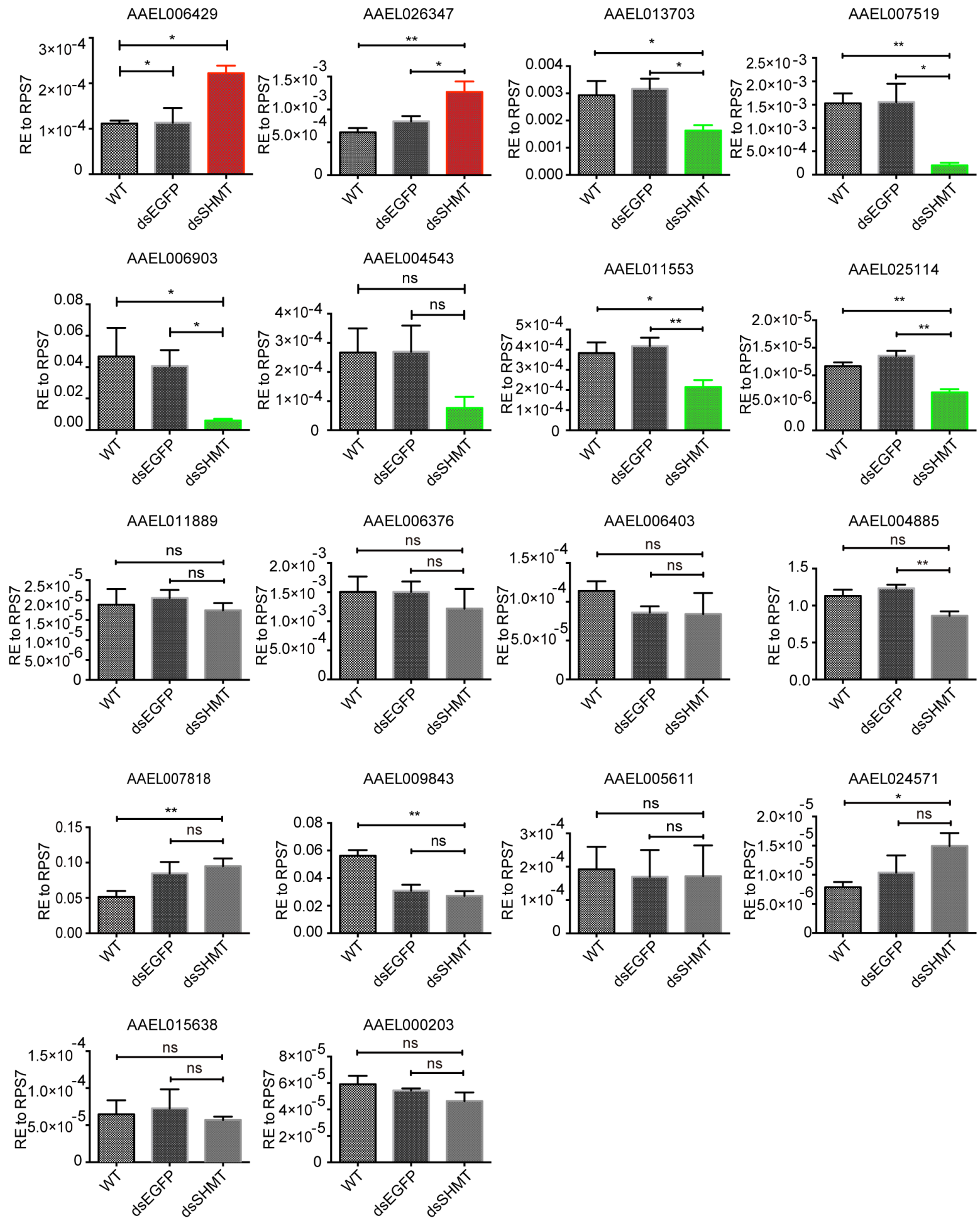


**Figure S2.** Transcriptional expression of trypsins responding to *SHMT* RNAi*.* The data are shown as the mean ± SEM. **P* < 0.05; ***P* < 0.01; ns, not significant.


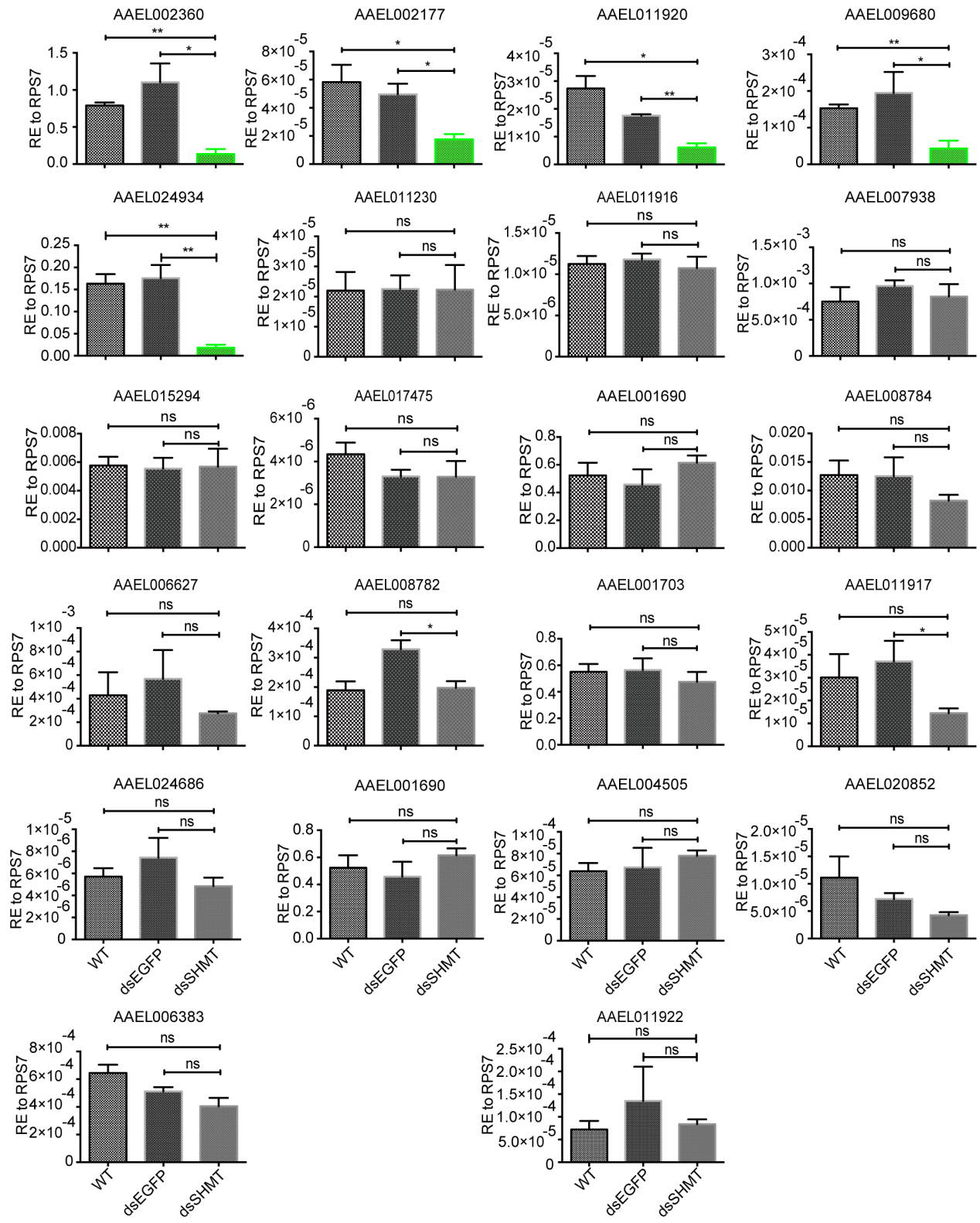


**Figure S3.** Transcriptional expression of chymotrypsins responding to *SHMT* RNAi. All graphs have the same abscissa, i.e. three samples, WT, dsEGFP and dsSHMT. The data are shown as mean ± SEM. **P* < 0.05; ***P* < 0.01; ns, not significant.


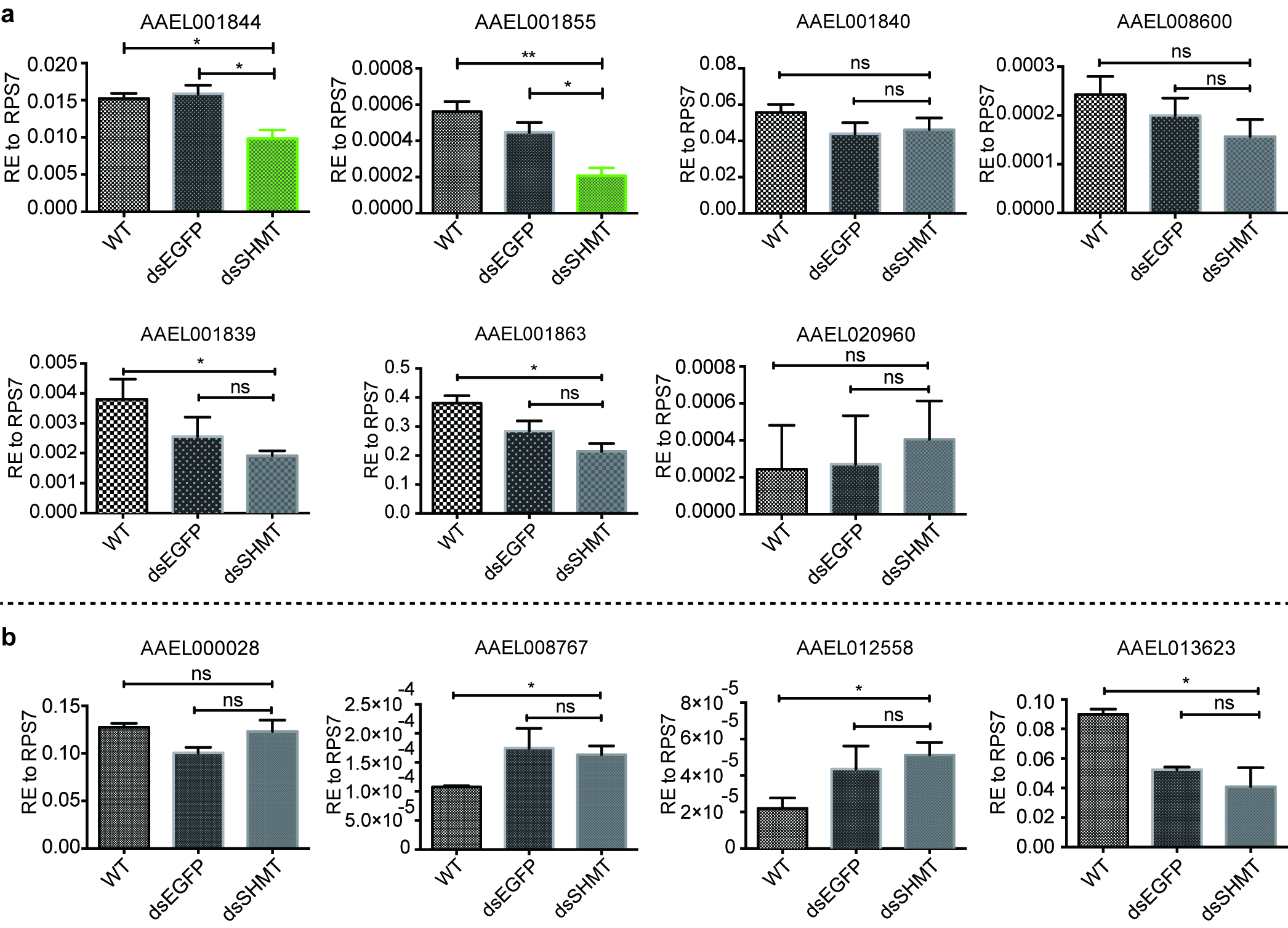


**Figure S4.** Transcriptional expression of carboxypeptidases and serine proteases responding to *SHMT* RNAi. **a** Transcriptional expression of carboxypeptidases. **b** Transcriptional expression of serine protease. The data are shown as the mean ± SEM. **P* < 0.05.
